# THE MODERATING ROLE OF RACE AND ETHNICITY BETWEEN NEGATIVE FAMILY INTERACTIONS AND MENTAL HEALTH AMONG OLDER ADULTS

**DOI:** 10.1093/geroni/igad104.0802

**Published:** 2023-12-21

**Authors:** Fei Wang, Ann nguyen, Karen Lincoln, Weidi Qin, Tyrone Hamler

**Affiliations:** Case Western Reserve University, Cleveland, Ohio, United States; Jack, Joseph, and Morton Mandel School of Applied Social Sciences, Case Western Reserve University, Cleveland, Ohio, United States; University of California, Irvine, Irvine, California, United States; University of Michigan, Ann Arbor, Michigan, United States; University of Denver, Denver, Colorado, United States

## Abstract

Studies generally show that negative social interactions are detrimental to mental health for older adults. Furthermore, empirical evidence suggests that negative interactions may function differently in relation to mental health across racial/ethnic groups given their unique life circumstances and social conditions. This study examines whether the association between negative family interactions and mental health outcomes varies by race and ethnicity. Samples of older African Americans, Caribbean Blacks, and non-Latino Whites aged 55 and older were drawn from the National Survey of American Life (N = 1,439). Mental health variables included depressive symptoms, any lifetime disorder according to The Diagnostic and Statistical Manual of Mental Disorders, fourth edition (DSM-IV), and number of lifetime DSM-IV disorders. Regression models were used to test the study aim. Analyses indicated that negative interactions with family were positively associated with all 3 mental health outcomes. Several racial/ethnic differences emerged. The association between negative family interactions and depressive symptoms was stronger among Whites than African Americans. While negative family interactions were positively associated with number of disorders among Caribbean Blacks, negative interactions were unrelated to number of disorders among African Americans. This study demonstrates the racial and ethnic differences in diverse aging populations and the importance of recognizing the heterogeneity of the Black American population in minority research. Clinical practice should focus on reducing negative family interactions, and future research should examine whether psychosocial resources (e.g., stress appraisals, neighborhood social cohesion) can attenuate the association between negative family interactions and mental health for older African Americans.

